# Posttranslational Modifications of Pyruvate Kinase M2: Tweaks that Benefit Cancer

**DOI:** 10.3389/fonc.2018.00022

**Published:** 2018-02-07

**Authors:** Gopinath Prakasam, Mohammad Askandar Iqbal, Rameshwar N. K. Bamezai, Sybille Mazurek

**Affiliations:** ^1^School of Life Sciences, Jawaharlal Nehru University, New Delhi, India; ^2^Department of Biotechnology, Faculty of Natural Sciences, Jamia Millia Islamia, New Delhi, India; ^3^Institute of Veterinary Physiology and Biochemistry, University of Giessen, Giessen, Germany

**Keywords:** M2 isoform of pyruvate kinase, posttranslational modifications, cancer metabolism, glycolysis, Warburg effect, cancer therapy

## Abstract

Cancer cells rewire metabolism to meet biosynthetic and energetic demands. The characteristic increase in glycolysis, i.e., Warburg effect, now considered as a hallmark, supports cancer in various ways. To attain such metabolic reshuffle, cancer cells preferentially re-express the M2 isoform of pyruvate kinase (PKM2, M2-PK) and alter its quaternary structure to generate less-active PKM2 dimers. The relatively inactive dimers cause the accumulation of glycolytic intermediates that are redirected into anabolic pathways. In addition, dimeric PKM2 also benefits cancer cells through various non-glycolytic moonlight functions, such as gene transcription, protein kinase activity, and redox balance. A large body of data have shown that several distinct posttranslation modifications (PTMs) regulate PKM2 in a way that benefits cancer growth, e.g., formation of PKM2 dimers. This review discusses the recent advancements in our understanding of various PTMs and the benefits they impart to the sustenance of cancer. Understanding the PTMs in PKM2 is crucial to assess their therapeutic potential and to design novel anticancer strategies.

## Introduction

The key metabolic anomaly associated with tumor cells in contrast to their non-transformed counterparts is rewiring of metabolism to escalate glucose uptake rate and to break down glucose largely into lactate, regardless of the presence of oxygen, a phenomenon known as aerobic glycolysis or Warburg effect (1). The preferential expression of M2 isoform of pyruvate kinase (PKM2) by the cancer cells is required for metabolic reprogramming, a nearly universal phenomenon in cancer (2–6). To further ripe benefits out of the PKM2 expression, cancer cells posttranslationally modify it into an enzymatically less active dimeric state. Thus, retarding the PKM2 catalyzed conversion of phosphoenol pyruvate (PEP) into pyruvate, causing piling up of the glycolytic intermediates and their channeling into pentose phosphate pathway (PPP) as well as phospholipid and amino acid synthesis to facilitate biosynthesis of macromolecules and reducing equivalent [nicotinamide adenine dinucleotide phosphate (NADPH + H+)], eventually supporting cancer cell proliferation (4). The dimer and tetramer ratio of PKM2 is regulated by numerous factors including metabolic intermediates [fructose 1, 6-bisphosphate (FBP), succinylaminoimidazolecarboxamide ribose 5-phosphate (SAICAR) and serine], physical interaction with oncoproteins, and importantly by various posttranslation modifications (PTMs) (7–9). Notably, the PTMs contribute to expansion of functional proteome in the mammalian cells (10). Recent advancements in PTMs research have uncovered many intricate regulatory mechanisms through which PTMs regulate the cell signaling and metabolic pathways. PKM2 protein sequence consists of many conserved amino acid sites that are modified covalently by PTMs such as phosphorylation, acetylation, hydroxylation, oxidation, ubiquitination, and sumoylation. This review deals with investigating the extrinsic and intrinsic stimuli responsible for PTMs of PKM2 and scrutinizes the impact on PKM2 biology along with the associated canonical and non-canonical functional implications that benefit cancer.

## Mammalian PK Gene, Transcript Variants, and Proteins

Pyruvate kinase (ATP-pyruvate 2-O-phosphotransferase, EC 2.7.1.40), a terminal glycolytic pathway enzyme, catalyzes the irreversible transphosphorylation reaction between PEP and ADP to yield a molecule of pyruvate and ATP in an oxygen-independent manner. All living entities ubiquitously express enzyme PK, thereby conserving at least one form of PK. The human genome consists of two PK genes; the *PKLR* gene on chromosome 1 and the *PKM* gene on chromosome 15, which code for four PK isoforms designated as PKL, PKR, PKM1, and PKM2 (11). The expression of PK isoforms is highly regulated and is tissue-specific, suggesting that different kinetic properties of PK isoforms satisfy different metabolic needs of different tissues. For instance, PKM2 is predominantly expressed in the tissues with high anabolic biosynthesis such as proliferating embryonic cells and tumor cells. Likewise, tissues bearing a high rate of gluconeogenesis, such as liver, possess accordingly compatible PKL isoform. The *PKLR* gene, by using tissue-specific alternate promoters, codes for the full-length PKR isoform in erythrocytes and a shorter variant PKL isoform (lacking 1 exon) in the liver as well as to a smaller extent in intestine and kidney (12). The *PKM* gene codes for PKM1 and PKM2 isoforms, through alternative splicing of the mutually exclusive exons 9 and 10. Out of 12 exons of PKM pre-mRNA, the mature transcript that includes exon 9 and excludes exon 10 is designated as PKM1. The mature mRNA that includes exon 10 and excludes exon 9 is termed PKM2 (M2-PK). The proteins coded by these mature mRNAs’ are of identical size (531 amino acids); however, out of 56 amino acids that are coded by the exon 9 and exon 10, 22 amino acids are different (13). The expression of the constitutively active PKM1 isoform is ideal for adult differentiated tissues that demand a large supply of ATP such as brain, adult skeletal muscle, and heart. The PKM2 isoform is mainly expressed in the dividing cells with growing anabolic demands, e.g., embryonic cells and cancer cells (5, 13).

The prototypic PKM2 isoform is highly expressed in embryonic tissues and is steadily replaced by other isoforms during differentiation. There are several studies substantiating the isoform shift back to PKM2 during tumorigenesis. For instance, L-M2 shift in hepatocarcinogenesis (14, 15), M1–M2 shift during metabolic transformation, i.e., aerobic glycolysis (16). Although M2 is tightly associated with tumor growth and metabolism (as suggested by the reduction in tumor growth on replacing M2 by M1), it is also present in some differentiated tissues such as lung, adipose, pancreatic islets, retina, distal renal tubules, and so on (17, 18).

## Regulation of PKM2 Re-Expression

The molecular mechanism that regulates the alternative splicing of PKM isoforms remained ambiguous until recently. The study by David et al. unraveled a potential mechanistic link by which cancer cells could re-express PKM2. In this study, the authors revealed the role of c-Myc (the most frequent deregulated oncogene)-mediated transcriptional activation of heterogeneous nuclear ribonucleoproteins [hnRNPA1, hnRNPA2, and polypyrimidine tract binding protein (PTB)] in regulating alternative splicing of the PKM isoforms (19). The hnRNPs’ selectively bind the sequence flanking exon 9 in PKM pre-mRNA to repress its inclusion to the mature mRNA, and thus, indirectly facilitating the inclusion of exon 10 into the mature mRNA, resulting in the expression of PKM2 mRNA in cancer cells.

The expression of PKM2 has been shown to be influenced by a spectrum of signaling pathways that are stimulated by tumor microenvironment (hypoxia and nutrient status), aberrant oncogenic mutations, growth factors, and hormones. These signaling pathways, through the network of transcription factors, regulate PKM2 expression. Transcription factors that directly bind to the consensus sequence in the *PKM* gene include hypoxia-inducible factor-1α (HIF-1α), nuclear factor kappa B (NF-κB), peroxisome proliferator-activated receptor gamma (PPARγ), and specificity protein 1 (Sp1) (8, 20). Under hypoxic conditions, HIF-1 stimulates the expression of PKM2 isoform by binding to the hypoxic response element present in the first intron of PKM gene. Notably, PKM2 interacts with HIF-1 transcription complex to co-regulate its own transcription through a positive feedback loop (21). In addition to hypoxia, aberrant oncogenic signaling in cancer cells has been shown to facilitate the normoxic stabilization of HIF-1α to rewire the metabolism by inducing the expression of PKM2. For example, mammalian target of rapamycin (mTOR), downstream to the axis of receptor tyrosine kinase/PI3K/protein kinase B (AKT), which is frequently deregulated in cancer cells, is known to stimulate the expression of c-Myc and normoxic stabilization of HIF-1. Both transcription factors increase the transcriptional activation of many glycolytic enzymes including PKM2 to stimulate aerobic glycolysis (22). Accordingly, the stimulation of the PI3K/AKT/mTOR pathway by insulin facilitated the normoxic stabilization of hypoxic inducible factor 1 (HIF1), thereby increasing PKM2 expression to promote cancer type metabolism (23). Following EGFR stimulation, NF-κB (p65) induces the expression of PKM2 by recruiting HIF-1α to co-regulate the transcriptional activation of the *PKM* gene (24). In PTEN null mouse liver cells, hyperactivated AKT2 induced the expression of peroxisome proliferator-activated receptor gamma (PPARγ), a nuclear hormone receptor and a transcription factor. PPARγ specifically binds to the promoter of the *PKM* and *HK* (hexokinase) genes to augment their expression, thus causing the metabolic reprogramming which contributes to the liver pathophysiology (25). Hormones, such as insulin (23, 26, 27), triiodothyronine-T3 (28), and glucocorticoid (29) also regulate PKM2 gene expression. The Sp1 transcription factor constitutively activates the transcription of the *PKM* gene by binding to the consensus DNA binding sites (GC boxes) in the PKM gene promoter. The Sp3 transcription factor synergies Sp1 to enhance the abovementioned function (30). Glucose stimulation facilitates dephosphorylation of Sp1 by protein phosphatase 1, which enhances its DNA binding activity (GC boxes). As a result demonstrates an increased PKM transcription (31).

In addition to the abovementioned mechanisms, PKM2 gene expression is also enhanced by epigenetic hypomethylation of intron 1 within the PKM gene (32). miRNAs including miR-122, miR-133a, miR-133b, and miR-326 also affect the expression of PKM2 and thus metabolic reprogramming in cancer (33–36). In conclusion, the plasticity in the regulation of the PKM gene expression is exploited by cancer cells to promote their growth and metabolism.

## Structural Biology of PKM2

**Figure 1:**
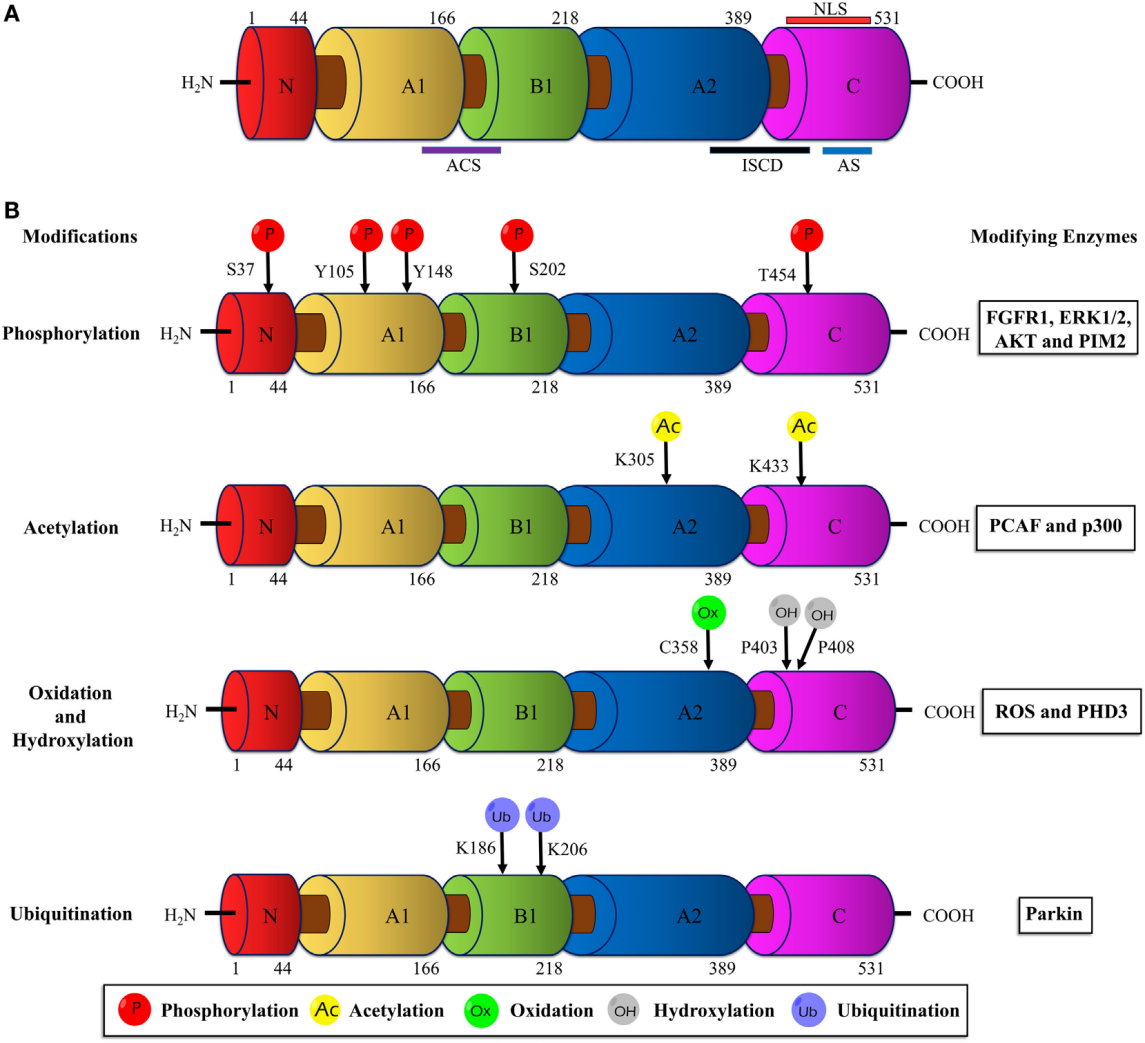
Schematic illustration of the PKM2 domain structures and major sites of posttranslational modifications (PTMs). **(A)** The interface between the A1 and B domains of PKM2 forms the catalytic active site (ACS); the flanking region between the A2 and C domains, known as intersubunit contact domain (ISCD), is a key component that enables the formation of tetrameric oligomers; the C domain accommodates the allosteric activator (FBP) binding site and a nuclear localization signal sequence (NLS). **(B)** The major sites of PKM2 that are subjected to phosphorylation, acetylation, oxidation, hydroxylation, and ubiquitination are marked and the modifying enzymes or factors that dictate the PTMs are added on the right. Abbreviations: PK, pyruvate kinase; p, phosphorylation; Ac, acetylation; Ox, oxidation; OH, hydroxylation; Ub, ubiquitination; FGFR1, fibroblast growth factor receptor-1; ERK1/2, extracellular signal-regulated kinase 1/2; AKT, protein kinase B; p300, histone acetyltransferase; ROS, reactive oxygen species; PHD3, prolyl hydroxylase 3; PKM2, M2 isoform of pyruvate kinase; FBP, fructose 1, 6-bisphosphate.

Human PKM2 protein comprises 531 amino acids and each monomeric subunit is subdivided as A-, B- and C domains based on their characteristic functional features (Figure 1A). The adjoining region between the A- and B domains together forms the catalytic active site. The C domain near the carboxyl terminus harbors the binding site for the allosteric activator (FBP) as well as the intersubunit contact domain (ISCD) and the nuclear localization signal sequence. Notably, the 22 amino acids that differ between PKM1 and PKM2 (coded by the alternatively spliced exons) span across the ISCD domain (37). This difference allows PKM2 to have unique functional properties such as allosteric regulation by FBP and the ability to exist in tetrameric and dimeric forms. In contrast, PKM1 is not allosterically regulated and exists only in a tetrameric form.

The dimeric PKM2 emerges as the result of an interaction between two monomers at their A domain. Two PKM2 dimers associate at their C domain (ISCD) to form the tetrameric PKM2. In addition, PKM2 can form hybrids with PKL in liver, jejunum, colon, and rectum. Likewise, heterotetrameric hybrids between PKM2 and PKM1 were reported in esophagus, stomach, and also recently in lung and breast cancer cell lines (29, 38–40).

## Dynamic PKM2 Activity Regulation and Cancer Metabolism

Although the different isozymes of PK catalyze the identical biochemical reaction, they differ in their kinetic properties such as varied affinity to its substrate PEP, allosteric regulation, and ability to exist in more than one oligomeric state. Unlike PKM1 isoform, a constitutively active tetrameric enzyme, the activity of PKM2 is influenced by its oligomeric state, i.e., dimer or tetramer. Whereas the tetrameric form of PKM2 has a high affinity for PEP and is highly active the dimeric form is characterized by a low PEP affinity and is nearly inactive under physiological conditions (3). The less active dimeric form of PKM2 supports the channeling of glucose carbons into synthetic processes which debranch from glycolytic intermediates, while the active tetrameric PKM2 is relatively less supportive to anabolic synthesis. The tetramer:dimer ratio of PKM2 is regulated by different mechanisms, i.e., metabolic intermediates, binding of oncoproteins, and posttranslational modifications (5).

The glycolytic intermediate FBP and the amino acid serine synthesized from glycolytic glycerate 3-P induce the subunit association from dimeric PKM2 to the enzymatically highly active tetramer (41, 42). In the same way, binding of SAICAR (an intermediate from the *de novo* purine biosynthesis pathway) to PKM2 stimulates PK activity under glucose-deprived conditions (43). PKM2 activity is allosterically inhibited by the amino acids—alanine, tryptophan, and phenylalanine (6, 8, 9). Besides the metabolic intermediates, numerous proteins are known to physically interact with PKM2 to modulate its enzymatic activity. The E7 oncoprotein encoded by the high-risk type human papillomavirus 16 binds to PKM2 to facilitate its subunit dissociation from tetramer to inactive dimer even in the presence of the allosteric activator FBP (44). Interaction of PKM2 with cytosolic promyelocytic leukemia (cPML), Jumonji C domain containing dioxygenase (JMJD5), and Tyr-46-phosphorylated MUC1-C (mediated by EGFR) inhibit PK activity through different mechanisms (45–47). cPML selectively binds the tetrameric PKM2 and inhibits its activity without dissociating it into PKM2 dimers. JMJD5 decreases PKM2 activity by inhibiting the formation of active tetramers. However, the underlying mechanism of inhibition of PK activity by Tyr-46-phosphorylated MUC1-C is not known. Interestingly, the interaction between PKM2 and death-associated protein kinase, a serine/threonine kinase enhances the enzyme activity of PKM2 (48). Thus, PKM2 acts as a molecular switch in regulating the metabolic fate of glycolytic intermediates (energy generation or anabolism) *via* modulation of its activity and structural configuration by the abovementioned mechanisms.

## Posttranslational Modifications of PKM2 and Implications in Cancer

Unlike other isoforms of PK, PKM2 harbors numerous conserved PTM sites, often modified by phosphorylation, acetylation, prolyl-hydroxylation, oxidation, ubiquitination, and sumoylation in response to various stimuli in cancer cells (Figure 1B). These PTMs modulate structural and functional properties of PKM2, such as oligomeric state, catalytic activity, binding of allosteric activators, protein stability, conditional protein interaction, and subcellular localization. Eventually, the posttranslationally modified PKM2 provides metabolic and non-metabolic benefits to cancer cells.

## Phosphorylation

### Tyrosine Phosphorylation

**Figure 2:**
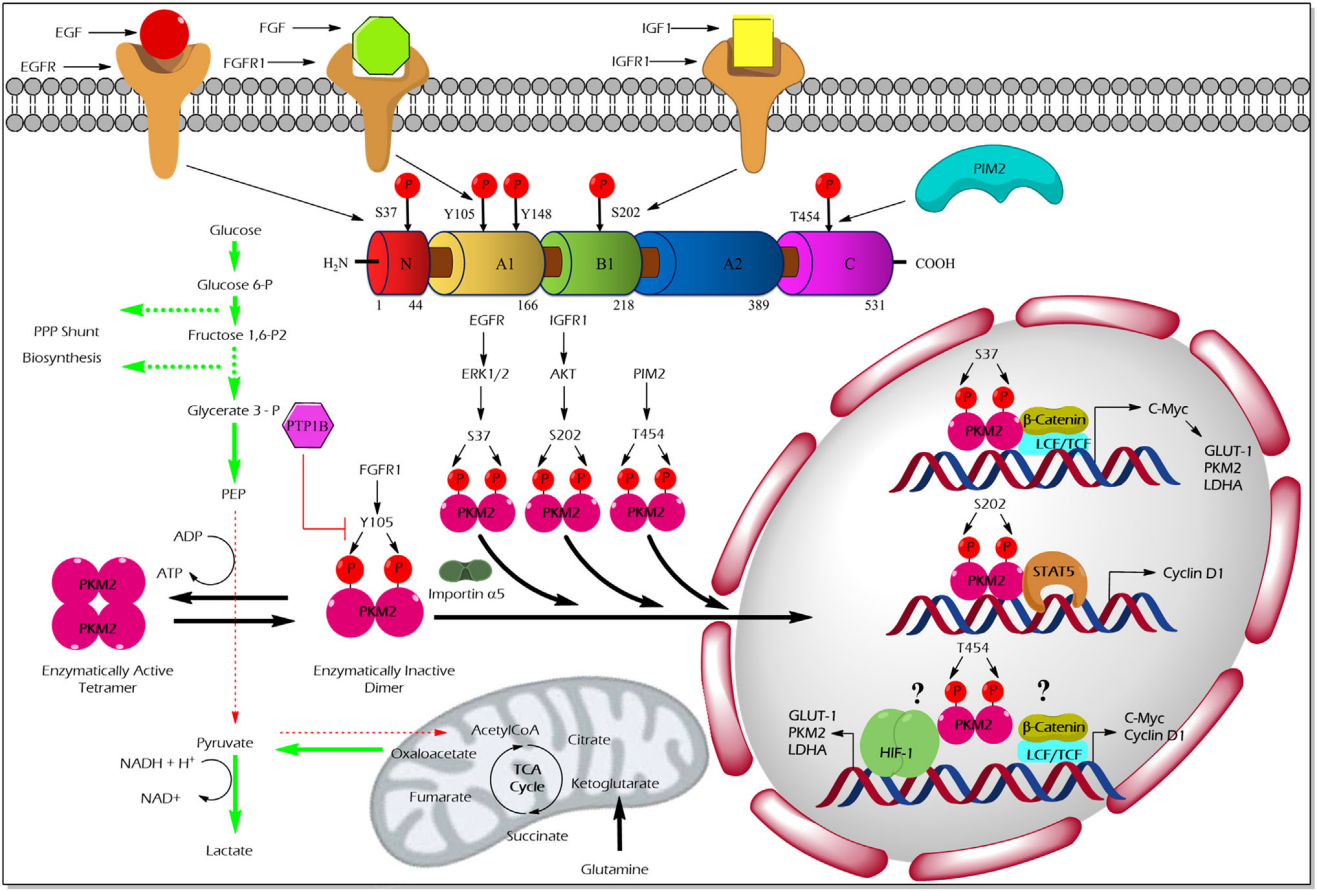
Regulation of M2 isoform of pyruvate kinase (PKM2) enzyme activity and non-glycolytic nuclear functions by phosphorylation. Aberrant oncogenic FGFR1-mediated tyrosine (Y) phosphorylation of PKM2 at Y105 residue facilitates the formation of the enzymatically inactive dimeric form of PKM2 which promotes aerobic glycolysis and biosynthesis of macromolecules by causing accumulation and diversion of the glycolytic intermediates into synthetic pathways of cell building blocks. Conversely, protein-tyrosine phosphatase 1B (PTP1B), a tyrosine protein phosphatase, dephosphorylates tyrosine phosphorylated PKM2 and reverses the above-stated features to establish metabolic homeostasis under physiological conditions. Unlike the Y105 phosphorylation, serine (S) or threonine (T) phosphorylation of PKM2 at S37, S202, and T454 by growth factor-stimulated serine/threonine protein kinases [extracellular signal-regulated kinase (ERK) 1/2, protein kinase B (AKT), and proviral insertion in murine lymphomas 2 (PIM2)], direct the nuclear translocation of PKM2 with the aid of nuclear importin α5. The nuclear-localized PKM2 assists the transcriptional activation of β-catenin and signal transducer and activator of transcription 5 (STAT5) to facilitate the expression of different genes such as cyclin D1, c-Myc, glucose transporter 1 (GLUT1), lactate dehydrogenase A (LDHA), and PKM2, all essential for tumor cell metabolic reprogramming and division. Green arrows in the illustration indicate the augmented glycolytic flux. Red arrows indicate the restricted flux of glycolysis and the fate of the glycolytic intermediates as a result of the dimeric state of PKM2.

The pioneering study from the laboratory of Eigenbrodt et al.demonstrated that Src—non-receptor tyrosine kinase—phosphorylates PKM2 to inhibit its catalytic activity (49). Further studies by his team suggested that tyrosine phosphorylation results in an inhibition of PKM2 thereby supporting the diversion of the glycolytic flux into synthetic routes of cell building blocks (2, 50). Nearly two decades later, Hitosugi et al. confirmed the findings and demonstrated that aberrant oncogenic tyrosine kinases (e.g., FGFR1, BCR-ABL, and JAK2) phosphorylate tyrosine (Y) 105 residue of PKM2 to inhibit its activity. They further showed that Y105 phosphorylation hinders the allosteric activation of PKM2 by FBP, resulting in PKM2 activity inhibition and channeling of glycolytic intermediates into biosynthetic metabolism to support tumor growth (4) (Figure 2). Hitosugi et al., using a phosphoproteomic screen, also identified additional phosphorylated tyrosine residues in PKM2 including, Y83, Y148, Y175, Y370, and Y390. However, subsequent characterization using mutation analysis revealed that only Y105 phosphorylation is significant in inhibiting PKM2 activity and in supporting tumor metabolism and growth. The dynamic regulation of PKM2 Y105 phosphorylation/dephosphorylation may result in a fine-tuning between aerobic glycolysis and oxidative phosphorylation to support distinct metabolic needs of the proliferating and quiescent cells. The role of tyrosine phosphatases that dephosphorylate PKM2 to rescue its activity remained unclear. Recent studies on adipocytes, using substrate trapping and mutagenesis assay, revealed an interaction between protein-tyrosine phosphatase 1B and the tyrosine 105 domain of PKM2 thereby inducing its dephosphorylation (51) (Figure 2).

Apart from tyrosine phosphorylation, PKM2 has been shown to specifically bind tyrosine-phosphorylated peptides or proteins, which in turn force PKM2 to release its allosteric activator FBP, leading to an inhibition of PKM2 activity, and thus, supporting anabolic metabolism in dividing tumor cells (52). Yang et al. reported that the epidermal growth factor (EGF) stimulated nuclear translocation and association of PKM2 with Y333 phosphorylated β-catenin. This interaction mediates the binding of the β-catenin/TCF transcription complex to the CCDN1 promoter responsible for the co-activation of cyclin D1 transcription, thus, promoting brain tumor development (53).

### Serine/Threonine Phosphorylation

Unlike tyrosine phosphorylation of PKM2 that principally modulates its glycolytic function, serine/threonine phosphorylation of PKM2 supports its nuclear localization and opens up new vistas of non-glycolytic moonlight functions, i.e., gene transcriptional regulation and protein kinase activity (Figure 2).

Cancer cells stimulated with EGF triggers extracellular signal-regulated kinase 2 (ERK2), serine/threonine protein kinase, to phosphorylate PKM2 at the serine (S) 37 residue. PIN and importin α5 selectively bind the S37-phosphorylated PKM2 and induce its translocation into the nucleus where PKM2 serves as a transcriptional co-activator of β-catenin to express its target gene, c-Myc. The transcription factor c-Myc, in turn, enhances the expression of glucose transporter 1 (GLUT1), lactate dehydrogenase A, and remarkably PKM2, through a positive feedback loop. Together, EGF-stimulated S37 phosphorylation of PKM2 facilitates metabolic reprogramming in cancer cells (54). Likewise, an extracellular matrix protein 1 (a secretory glycoprotein) has been shown to stimulate PKM2 S37 phosphorylation, to enhance the transcriptional activation of glycolytic enzymes that support aerobic glycolysis in tumor cells (55). AKT stimulated by insulin-like growth factor (IGF-1) in cancer cells has been shown to interact and phosphorylate PKM2 at the S202 residue to promote its entry into the nucleus to facilitate the transcriptional activation of signal transducer and activator of transcription (STAT)5A targets gene, cyclin D1 (56). This study proposes PKM2 as a co-activator of the transcription factor, STAT5A, which functions downstream to the IGF/PI3K/AKT signaling pathway to promote tumor growth (56). The effect of PKM2 serine phosphorylation by A-Raf is dependent upon the metabolism of serine, alanine, and glutamine. For instance, primary fibroblasts which are characterized by serine consumption and glutamine production, expression of wild-type A-Raf induces PKM2 dimerization which leads to inhibition of the metabolism of glucose to lactate. On the other hand, NIH 3T3 cells, characterized by glutamine consumption and serine production, expression of gag-A-Raf favors the metabolism of glucose to lactate by increasing highly active tetrameric form of PKM2 (57). Oncogenic proviral insertion in murine lymphomas 2 (a serine/threonine protein kinase) has been shown to phosphorylate the threonine (T) 454 residue of PKM2 to stimulate its non-glycolytic nuclear function. The non-glycolytic function of T454 phosphorylated PKM2, primarily involves the transcriptional co-activation of HIF-1α and β-catenin transcription factors to support cancer growth and drug resistance (58).

## Acetylation

Acetylation is another important PTM where the class of enzymes termed N-terminal- or lysine-acetyltransferase catalyzes the transfer of the acetyl group from acetyl-CoA to the alpha- or epsilon-amino group of the lysine residue. Many basic cellular functions, such as chromatin remodeling, gene expression as well as several protein functions, and to maintain cellular homeostasis, depend upon an exact regulation of the dynamic process between acetylation and deacetylation.

**Figure 3:**
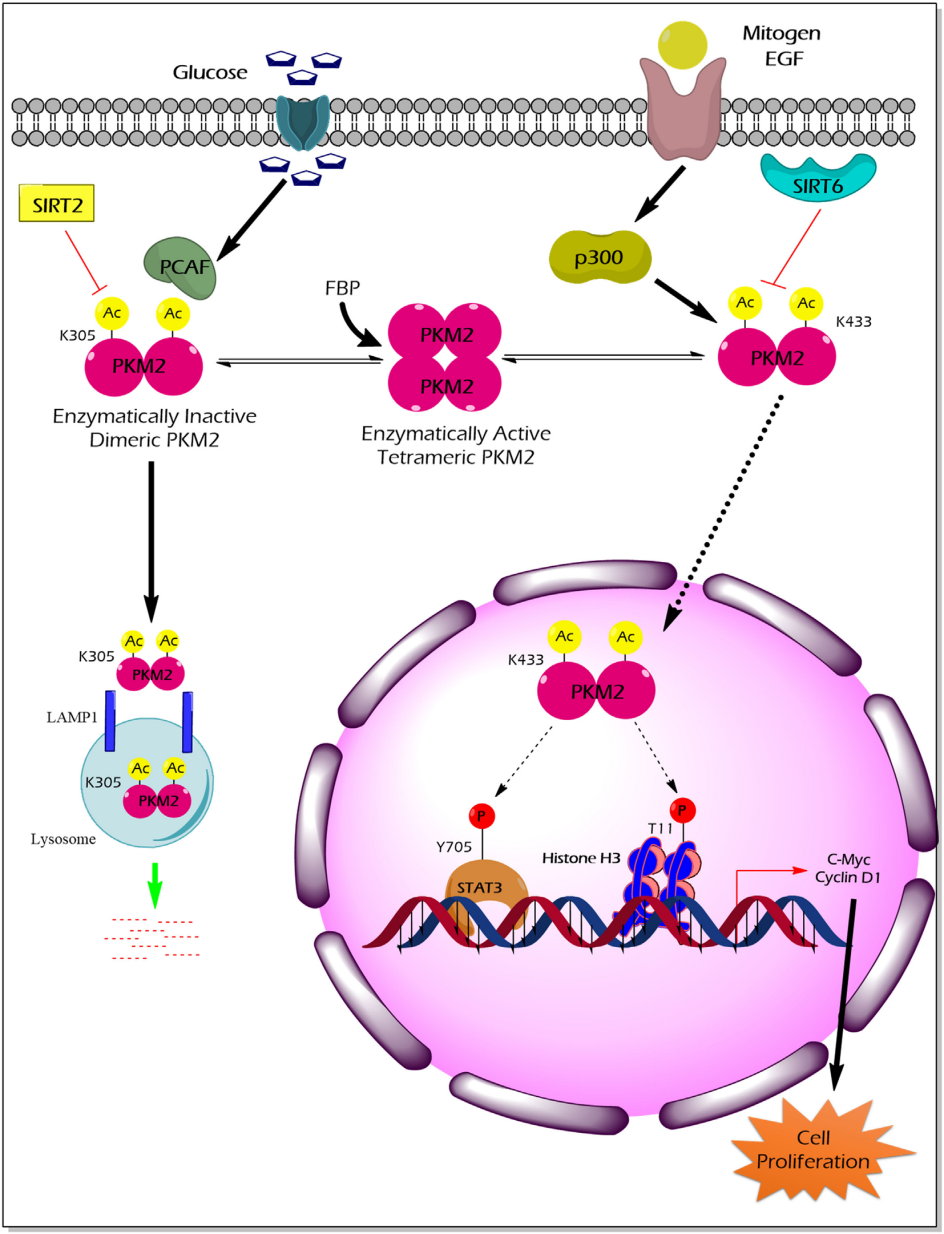
The impact of PKM2 acetylation on its enzymatic activity, protein stability, nuclear localization, and non-glycolytic functions. High glucose concentration facilitates the acetylation of PKM2 lysine (K) 305 residue by PCAF acetyltransferase. K305-acetylated PKM2 exhibits reduced enzyme activity and undergoes lysosomal degradation through chaperon-mediated autophagy. Low PKM2 expression and reduced enzyme activity support the anabolic metabolism and rapid division of tumor cells. Mitogens and oncogenic signals elicit acetyltransferase-p300 to acetylate K433 residues of PKM2, resulting in the release of the allosteric activator FBP from the PKM2 protein, translocation of PKM2 into the nucleus, and activation of the protein kinase activity of PKM2. Inside the nucleus, K433-acetylated PKM2 protein kinase phosphorylates STAT3 at the Y705 residue and histone H3 at T11, thereby activating the transcriptional program that the supports cell proliferation and tumorigenesis. SIRT2 deacetylates K305 residue, and SIRT6 deacetylates K433 residue of PKM2, thereby impairing the tumor-promoting property of PKM2. Abbreviations: EGF, epidermal growth factor; PCAF, P300/CBP-associated factor; p300, histone acetyltransferase; Ac, acetylation; SIRT2, NAD-dependent deacetylase sirtuin-2; SIRT6, NAD-dependent deacetylase sirtuin-6; FBP, fructose 1, 6-bisphosphate; STAT3, signal transducer and activator of transcription 3; LAMP1, lysosomal-associated membrane protein 1; PKM2, M2 isoform of pyruvate kinase.

Acetylation of PKM2 lysine residues 305 and 433 alter its activity, protein stability, and non-glycolytic protein kinase function (Figure 3). High glucose concentration induces lysine (K) 305 acetylation, mediated by the PCAF (P300/CBP-associated factor) acetyltransferase, in cancer cells to reduce PK activity. Lysine 305 acetylation targets PKM2 for lysosomal degradation through a chaperon-mediated autophagy. Hence, the state of reduced PKM2 expression and activity stockpiles the upper glycolytic intermediates and redirects the flux through the PPP shunt for biosynthesis of building blocks (nucleotides and amino acids), which eventually promotes tumor growth (59). Unlike the molecular mechanism described earlier, a recent study by Lv et al. revealed a remarkable non-glycolytic protein kinase function of PKM2 following its K433 acetylation mediated by p300 acetyltransferase in response to a spectrum of mitogenic and oncogenic stimuli (60). In detail, K433 acetylation of PKM2 promotes nuclear localization and protein kinase activity of the enzymatically inactive dimeric PKM2 to phosphorylate STAT3 at the Y705 residue and histone H3 at the T11 residue. The latter activates a transcriptional program that supports cell proliferation and tumorigenesis (60). Different deacetylases specifically remove the acetylation mark from PKM2 and limit the ability of PKM2 to promote tumorigenesis. For example, deacetylation of nuclear PKM2 by SIRT6 (sirtuin 6) at the K433 residue results in the export of PKM2 to the cytoplasm, thereby restricting its non-glycolytic functions (i.e., transcriptional co-activator and protein kinase) that support cancer (61). SIRT2 averts the metabolic benefits of PKM2 to cancer by deacetylation of PKM2 at the K305 residue which prevents the lysosomal degradation of PKM2 (62).

## Hydroxylation

In hydroxylation, proline or lysine amino acid residues of mammalian proteins are covalently modified by the addition of a hydroxyl group. Proline hydroxylation of mammalian proteins is primarily catalyzed by the prolyl hydroxylases family of enzymes in the presence of molecular oxygen, Fe^2+^ (iron), 2-oxoglutarate, and ascorbate. Proline hydroxylation has an important role in numerous cellular mechanisms, including cancer. A well-known example is the regulatory role of proline hydroxylation of the transcription factor HIF1 alpha depending upon oxygen supply.

**Figure 4:**
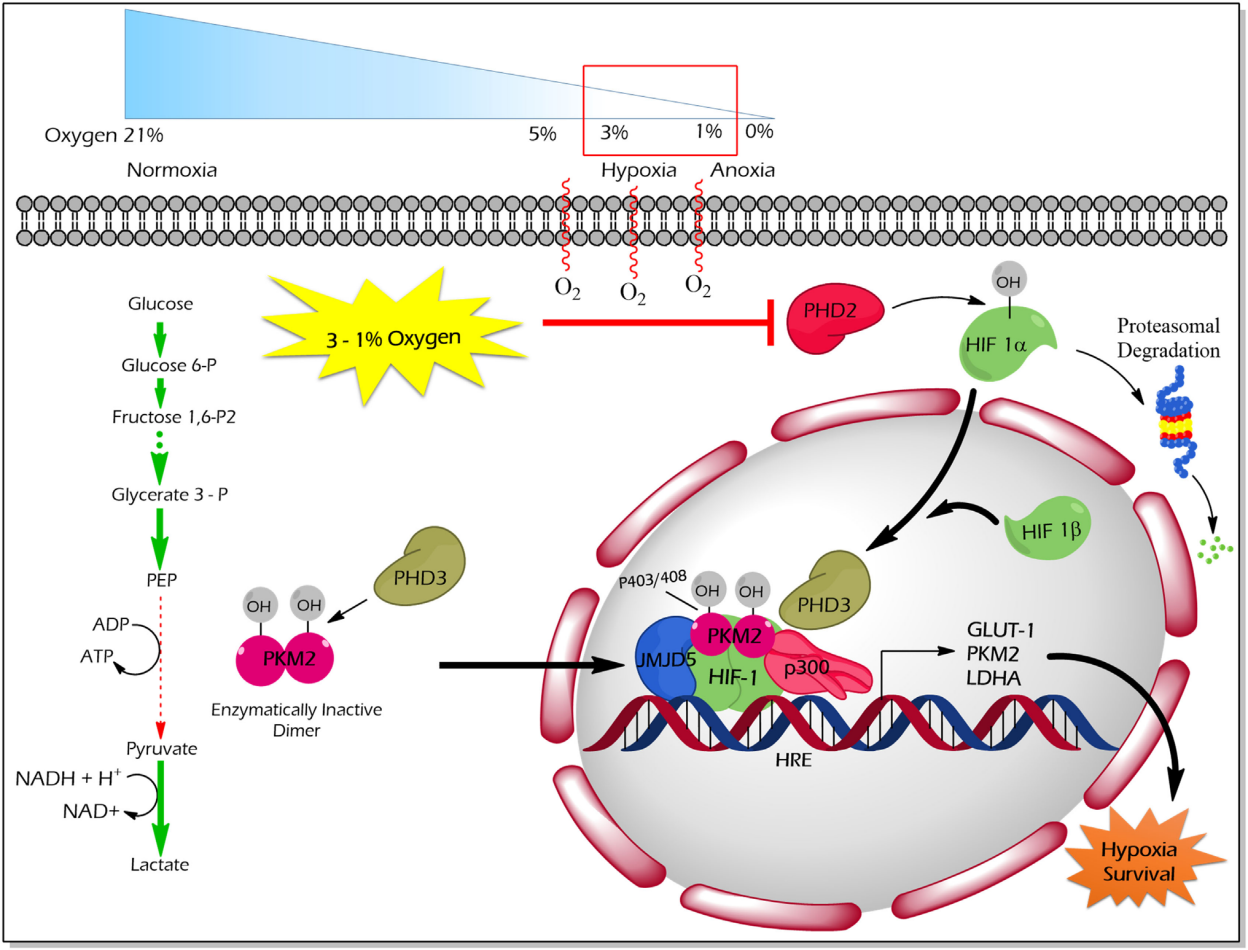
Regulation of non-glycolytic nuclear functions of PKM2 by proline hydroxylation. PHD3-dependent proline hydroxylated PKM2 (p402 and p408 residues) interacts with HIF-1 in the nucleus. PKM2 recruits p300 to HIF1 transcription complex and facilitate the transcription of target genes, which govern the metabolic reprogramming as well as the tolerance versus hypoxic condition. JMJD5 interaction with PKM2 assists the latter to localize into the nucleus and to promote HIF1-mediated transcriptional activation of glycolytic enzymes under hypoxic conditions. Abbreviations: OH, hydroxylation; HIF1, hypoxic inducible factor 1; PHD3, prolyl hydroxylase 3; JMJD5, Jumonji C domain-containing dioxygenase; p300, histone acetyltransferase; HRE, hypoxic response element; GLUT1, glucose transporter 1; LDHA, lactate dehydrogenase A; PKM2, M2 isoform of pyruvate kinase.

Proline (p) hydroxylation of PKM2 at 403 and 408 by the PHD3 enzyme (Figure 4) favors the interaction of PKM2 with the HIF1 transcription complex, which results in recruitment of p300-acetyltransferase to facilitate the transactivation of HIF target genes (21). Another study by Wang et al. revealed that under hypoxic conditions, JMJD5 interacts with PKM2 and facilitates its nuclear localization to promote HIF1 mediated transcriptional activation of glycolytic enzymes that support cancer cell metabolism and proliferation (47) (Figure 4).

## Oxidation

**Figure 5:**
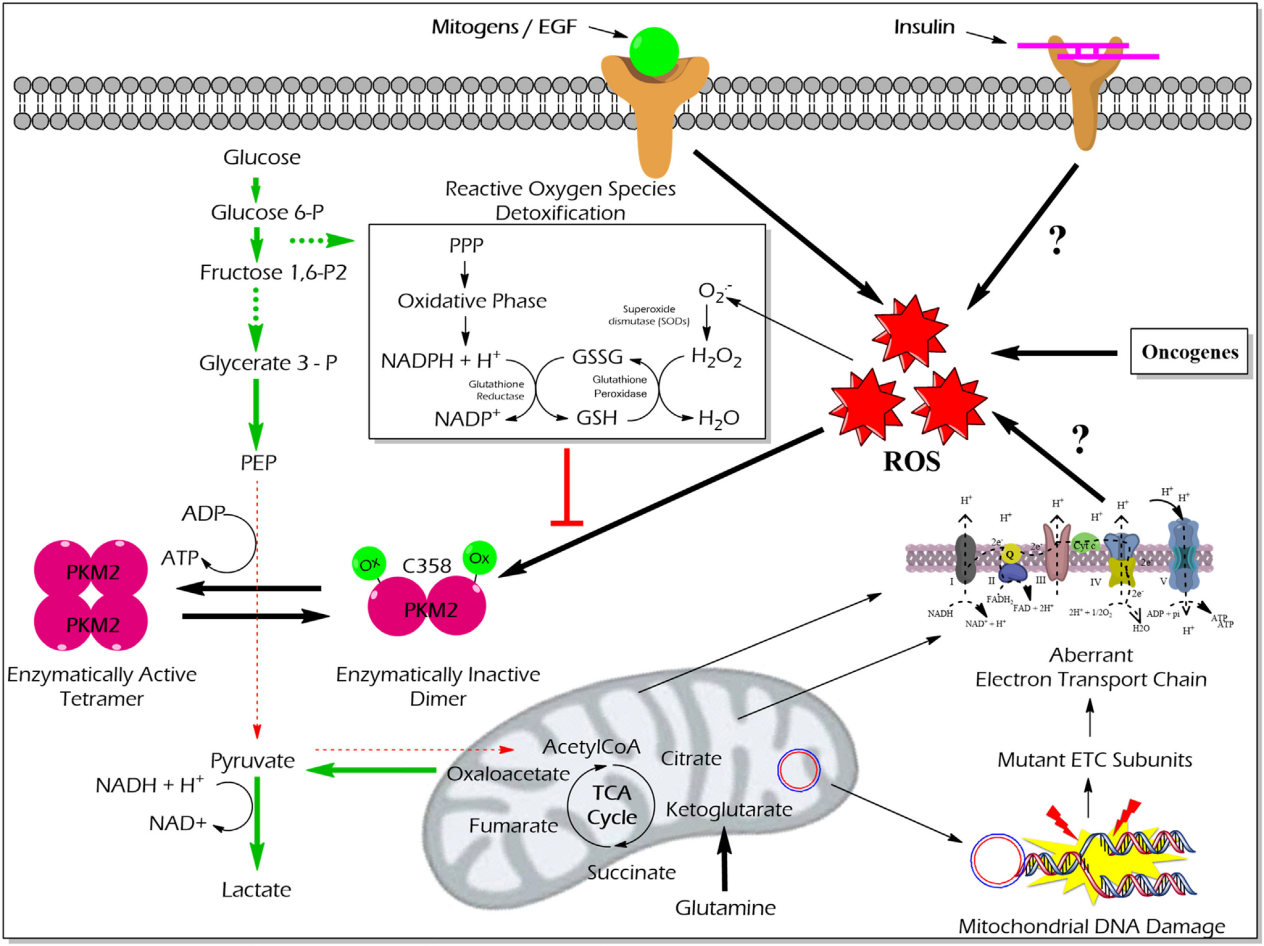
Effect of ROS-mediated oxidization of PKM2 on cellular metabolism and redox balance. Cancer cells produce large amounts of ROS that oxidize the cysteine (C) 358 residue of PKM2, which leads to the inhibition of PKM2 as well as a piling up of glycolytic intermediate glucose 6-phosphate which is then diverted into the PPP. The oxidative PPP facilitates the synthesis of NADPH + H^+^ which regenerates GSH thereby minimizing ROS-induced damages of the cancer cells. Abbreviations: ROS, reactive oxygen species; PPP, pentose phosphate pathway; NADPH, nicotinamide adenine dinucleotide phosphate; GSH, glutathione; GSSG, glutathione disulfide; H_2_O_2_, hydrogen peroxide; ETC, electron transport chain; PKM2, M2 isoform of pyruvate kinase; PPP, pentose phosphate pathway.

High reactive oxygen species (ROS) concentrations induce an oxidation of PKM2 at Cysteine 358 which results in a dissociation of PKM2 to the less active dimeric form. The functional characterization of PKM2 oxidation revealed an essential role in the conservation of the redox homeostasis in cancer cells. The enzymatically inert C358 oxidized PKM2 causes an accumulation of glycolytic intermediates which are required to channel glycolytic carbons into PPP thereby generating NADPH + H^+^ for detoxification of ROS (63) (Figure 5). Besides hypoxia and addition of either hydrogen peroxide or thiol oxidizing diamide into the cultivation medium of tumor cells also insulin is described to increase intracellular ROS production and cysteine oxidation of PKM2 (23).

## Ubiquitination and Sumoylation

M2 isoform of pyruvate kinase has been shown to interact with the HECT domain (a protein domain found in ubiquitin-protein ligases) of HERC1, a giant protein with a molecular weight of 532 kDa, which acts as both an E3 ubiquitin ligase and a guanine nucleotide exchange factor. The interaction between PKM2 and HERC1 neither altered PKM2 activity nor induced its proteasomal degradation (64). Since PKM2 also phosphorylates GDP, a possible role of PKM2 as a local GTP producer for the guanine nucleotide exchange function of the RLD1 (RCC1-like domain) of HERC 1 is discussed. Another E3-ligase which interacts with PKM2 is Parkin. Monoubiquitination of PKM2 and PKM1 at lysine (K) 186 and 206 residues by Parkin resulted in a reduction of PKM activity but did not affect its stability. The interaction between PKM2 and Parkin was favored by glucose starvation. Parkin silencing rescued the activity of PK and supported tumor growth (65). In addition, the laforin/malin E3-ubiquitin ligase complex, which is mutated in neurodegenerative Lafora disease, interacts and polyubiquitinates both M-isoforms of PK. When accessed for the biochemical properties, the enzyme activity of the PKM isoforms remained unaffected. Interestingly, this posttranslational modification impairs the nuclear localization of PKM2 following UV treatment. In the case of PKM1 neither UV treatment nor laforin/malin-mediated polyubiquitination affects its nuclear localization (66). Apart from interacting with E3 ubiquitin ligases, PKM2 does also bind the deubiquitin enzyme USP20 (ubiquitin-specific protease, a deubiquitin enzyme). USP20 interaction with PKM2 was suggested to apparently enhance its protein stability (67). The interaction of PKM2 with SUMO-E3 ligase PIAS3 (protein inhibitor of activated STAT3) induces sumoylation of PKM2 and translocation of the PKM2–PIAS3 complex into the nucleus. The functional significance of the interaction between PKM2 and PIAS3 as well as of the nuclear localization of the complex remains ambiguous (68). In summary, the functional relevance of the interaction of PKM2 with E3-ligases and deubiquitin enzymes as well as with SUMO E3-ligases is still not completely understood.

## Glycosylation

Threonine 405 and serine 406 residues of PKM2 were recently identified to be O-GlcNAcylated (*O*-linked β-*N*-acetylglucosamine) by O-GlcNAc transferase and this modification is dynamically regulated by the fluctuations in nutrient supply. Investigation of various human cancer cells and sporadic breast tumor tissue samples revealed PKM2 *O*-GlcNAcylation across the tumor cell types and patient samples. PKM2 T405 and S406 O-GlcNAc modification reduces PK activity by acting as a barrier that impedes the formation of stabilizing H-bonds at the C–C dimerization interface, thereby, destabilizing PKM2 active tetramer into enzymatically less active dimer. The state of reduced PK activity redirects the glycolytic metabolic intermediates to the anabolic metabolic pathway to support the synthesis of biomass and tumor proliferation. In addition, *O*-GlcNAcylation of PKM2 facilitates its translocation into the nucleus and the latter requires additional Ser37 phosphorylation of PKM2, mediated by EGF-stimulated ERK1/2 protein kinase and importin α5. O-GlcNAcylated, nuclear PKM2 activates transcription factor c-Myc to express GLUT1 and LHDA to further fine-tune aerobic glycolysis. The expression of *O*-GlcNAcylation-ablated PKM2 (T405A/S406A) reverses the pro-cancer metabolism and abrogates the tumor progression (69). Notably, this study highlights the crosstalk between PKM2 PTMs Thr405/Ser406 *O*-GlcNAcylation, and Ser37 phosphorylation is mutually inclusive to translocate PKM2 into the nucleus for executing non-glycolytic nuclear function (69).

## Methylation

Co-activator-associated arginine methyltransferase 1 also known as PRMT4 methylates specifically the dimeric form of PKM2 at Arg445/447/455 residues in the C domain. TEPP-46 and FBP, the allosteric activators that induce PKM2 tetramerization limits PKM2 methylation. Importantly, PKM2 activity remains unaltered by methylation; however, methylated PKM2 reprograms the metabolic phenotype toward aerobic glycolysis from oxidative phosphorylation to support tumor cell proliferation, migration, and metastasis. Mechanistically, methylated PKM2 localizes to the mitochondrial-associated endoplasmic reticulum membrane to interact with inositol 1, 4, 5-trisphosphate receptors (InsP_3_Rs) and to reduce InsP_3_R expression, thus, decreasing the mitochondrial membrane potential and Ca^2+^ uptake. This is essential to support TCA cycle and oxidative phosphorylation by activating the pyruvate dehydrogenase in Ca^2+^-dependent processes. The tumor supportive features of methylated PKM2 were averted by PKM2 knockdown or by introducing PKM2 methylation defective mutant or by delivering competitive non-methylated PKM2 peptide using nanoparticles (70).

## Concluding Remarks

The broad spectrum of PTMs in PKM2 which impart both metabolic and non-metabolic benefits to cancer underlines the significance of this isoenzyme in cancer cells. While PKM2 is expressed not only in tumor tissues but also in some differentiated cells and tissues, the posttranslational modifications of PKM2 are very specific for tumor cells. Accordingly, targeting the different posttranslational modifications of PKM2 is a promising strategy in cancer treatment (Table 1). Owing to the heterogeneous nature of tumors, it is important to expand our understanding of the roles of PTMs in PKM2, warranting further investigation and high-throughput PTM data from different tumors in order to refine the clinical targeting of PKM2 PTMs.

**Table 1:** The effect of various posttranslation modifications (PTM) of M2 isoform of pyruvate kinase (PKM2) on tumor metabolism and growth.

PTM	Modifying enzymes	Site of PTM	Effect on tumor metabolism and growth	Non-metabolic nuclear function	PKM2 knockdown (or) PTM-mimetic/ablative mutations	Reference
Tyrosine phosphorylation	pp60 v-src	Not characterized	Promotes aerobic glycolysis and tumor growth	Not characterized	Not characterized	(49, 50)
Tyrosine phosphorylation	FGFR1, BCR-ABL, and JAK-2	Tyr105	Promotes aerobic glycolysis and tumor growth	Not characterized	Regressed tumor growth	(4)
Serine phosphorylation	Extracellular signal-regulated kinase 2 and extracellular matrix protein 1	Ser37	Promotes aerobic glycolysis and tumor growth	Stimulates expression of c-Myc target genes [glucose transporter 1 (GLUT1) and lactate dehydrogenase A (LDHA)]	Regressed tumor growth	(54, 55)
Serine phosphorylation	AKT	Ser202	Promotes aerobic glycolysis and tumor growth	Stimulates expression of cyclin D1	Regressed tumor cell growth in culture	(56)
Threonine phosphorylation	Proviral insertion in murine lymphomas 2	Thr 454	Promotes aerobic glycolysis and tumor growth	Stimulates expression of hypoxia-inducible factor-1α (HIF-1α) and β-catenin target genes	Regressed tumor cell growth in culture	(58)
Lysine acetylation	PCAF	Lys305	Promotes aerobic glycolysis and tumor growth	Not characterized	PTM mimetic mutation (K305Q) promoted tumor growth	(59)
Lysine acetylation	p300 acetyl transferase	Lys433	Promotes aerobic glycolysis and tumor growth	Phosphorylates signal transducer and activator of transcription 3 and H3	PTM mimetic mutation (K433Q) promoted tumor growth and non-acetylated PKM2 (K433R) regressed tumor growth	(60)
Proline hydroxylation	Prolyl hydroxylases 3	Pro 403 and 408	Promotes aerobic glycolysis	Stimulates expression of HIF-1α target genes (GLUT1, PKM2 and LDHA)	No such assays were carried out	(21)
Cysteine oxidation	Reactive oxygen species	Cys358	Promotes aerobic glycolysis and tumor growth	Not characterized	Cysteine oxidation—ablative mutations (C358S) regressed tumor growth	(63)
Ubiquitination	Parkin	Lys186 and 206	Promotes aerobic glycolysis	Not characterized	Not elucidated	(65)
Serine/threonine O-linked glycosylation (*O*-GlcNAcylation)	O-GlcNAc transferase	Thr405 and Ser403	Promotes aerobic glycolysis and tumor growth	Stimulates expression of GLUT1 and LDHA	PTM-ablative mutations (T405A and S406A) regressed tumor growth	(69)
Arginine methylation	Co-activator-associated arginine methyltransferase 1	Arg 445/447/455	Promotes aerobic glycolysis and tumor growth	Not characterized	Regressed tumor growth by delivering competitive non-methylated PKM2 peptide using nanoparticles	(70)

## Author Contributions

GP, MI, RB, and SM equally participated in drafting the review article; RB and SM critically reviewed for important intellectual content; and GP designed all the graphics.

## Conflict of Interest Statement

The authors declare that the research was conducted in the absence of any commercial or financial relationships that could be construed as a potential conflict of interest. The reviewer LF and the handling editor declared their shared affiliation.
